# Advancing Type 1 Diabetes Treatment: 3 PM‐Oriented Innovations in Islet Transplantation and Biomaterial Engineering

**DOI:** 10.1002/dmrr.70215

**Published:** 2026-08-09

**Authors:** Fatemeh Sabet Sarvestani, Ramin Yaghobi, Mohsen Khosravi Maharlooei, Mehdi Golshan, Mohammad Hossein Karimi

**Affiliations:** ^1^ Transplant Research Center Shiraz University of Medical Sciences Shiraz Iran; ^2^ Biotechnology Incubator Shiraz University of Medical Sciences Shiraz Iran; ^3^ Department of Immunology, Biochemistry, and Molecular Biology Mayo Clinic Phoenix Arizona USA

**Keywords:** bioengineering, immunomodulatory, islets, predictive, preventive, and personalised medicine (3 PM), transplantation, type 1 diabetes

## Abstract

Type 1 Diabetes (*T1D*) is an autoimmune disorder marked by the immune‐mediated destruction of pancreatic β‐cells, resulting in insulin deficiency and dysregulated glucose control. Current therapies—such as insulin replacement and artificial pancreas systems—manage symptoms but fail to restore endogenous β‐cell function or halt disease progression. Transplantation strategies, including islet allo‐ and xenotransplantation, offer potential cures but remain limited by immune rejection, donor scarcity, and the adverse effects of systemic immunosuppression. Within the framework of predictive, preventive, and personalised medicine (3 PM), *T1D* management requires a paradigm shift towards early detection, targeted prevention, and individualised therapy. Predictive biomarkers and molecular phenotyping can identify high‐risk individuals and forecast graft outcomes, while preventive strategies—such as localised immunomodulation, bioenergetic support, and control of systemic inflammation—improve tolerance and graft longevity. Personalised interventions, including patient‐specific biomaterials, immunoprotective encapsulation, and regulatory T cell–based or stem‐cell–derived β‐cell replacement, address immune and metabolic heterogeneity. Recent progress in biomaterials, encapsulation, and 3D bioprinting enables the practical implementation of this 3 PM approach by enhancing oxygenation, vascular integration, and specific scaffold design. Integration of AI‐driven analytics, digital health monitoring, and multi‐modal diagnostics further supports predictive control of transplant outcomes. This review highlights advances in islet transplantation and regenerative biomaterial engineering as key enablers of the transition from reactive treatment to 3 PM‐guided, patient‐tailored therapy for *T1D*.

## Graphical abstract

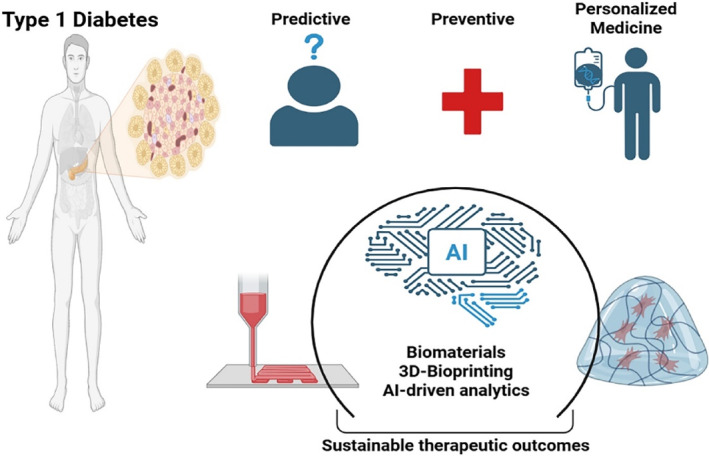

## 1 Introduction

Type 1 Diabetes (*T1D*) is a chronic autoimmune disorder characterised by the targeted, T–cell–mediated destruction of insulin‐producing pancreatic β‐cells, leading to impaired blood glucose regulation and the development of severe vascular and neuropathic comorbidities [1, 2]. Despite being one of the most extensively studied organ‐specific autoimmune diseases, efforts to prevent, halt, or reverse disease progression have failed to translate the promising results from animal models into consistent clinical success [3, 4]. The global incidence of *T1D* continues to rise by 3%–4% annually, with more than 542,000 children newly diagnosed each year, and over 40% of new cases now occurring in adults [2, 5, 6, 7]. Collectively, the direct and indirect economic burden of *T1D* exceeds $14 billion annually in the United States alone [8].

Although exogenous insulin therapy remains the cornerstone of treatment, it is far from curative. Patients continue to experience significant glycaemic fluctuations, recurrent episodes of hypo‐ and hyperglycemia, and a markedly increased risk of long‐term complications, including cardiovascular disease and chronic kidney disease [9, 10, 11, 12]. Even advanced technologies such as artificial pancreatic systems cannot fully replicate physiological glucose regulation. Similarly, β‐cell replacement strategies, via islet or whole‐organ transplantation, demonstrate potential but are constrained by donor shortages, invasive procedures, and the need for lifelong immunosuppression [13, 14, 15]. Immunosuppressive therapy itself provides only transient benefits, as autoimmunity frequently re‐emerges upon withdrawal and exposes patients to substantial risks such as opportunistic infections and malignancies [13].

From the perspective of predictive, preventive, and personalised medicine (3 PM), *T1D* exemplifies a condition in urgent need of paradigm‐shifting strategies. Three PM emphasises disease prediction using multilevel biomarkers, prevention by addressing early pathophysiology with targeted interventions, and personalisation through therapy selection guided by patient‐level phenotyping (immune, metabolic, organ‐specific, and digital). Embedding islet transplantation research within this framework requires (a) the development and reporting of predictive biomarkers that forecast graft outcome and disease progression, (b) preventive biomaterial and local immunomodulatory strategies that reduce or remove the need for lifelong systemic immunosuppression, and (c) personalisation of graft composition, implantation site, and adjunct immunotherapy according to patient‐specific profiles [16, 17, 18, 19, 20].

Recent advances in biomaterial science provide a unique opportunity to operationalise the principles of 3 PM in *T1D*. Beyond structural support, biomaterials are increasingly engineered to reduce antigen shedding, modulate local immune responses, and create favourable microenvironments for β‐cell survival and regulatory immune cell expansion [21, 22, 23]. These multifunctional materials, together with integrative immunotherapies, have the potential to transform disease management from reactive glucose control to predictive diagnostics, preventive interventions, and personalised regenerative solutions. This review, therefore, integrates biomaterial and transplantation advances with 3 PM concepts to explicitly demonstrate how the strategies discussed may contribute to the paradigm shift away from reactive care.

### 1.1 Preconditioning *T1D* Patients for Islet Transplantation: Chimerism and Thymic Tolerance

Although islet transplantation has demonstrated metabolic benefits, its widespread clinical application remains limited by alloimmune rejection and recurrent autoimmunity. Heterogeneity in autoimmune activity and systemic health influences both disease progression and transplant outcome. Inducing immune tolerance prior to transplantation is therefore a critical objective. Two promising immunological strategies to achieve this are the induction of mixed hematopoietic chimerism and the use of thymic‐based tolerance mechanisms [24, 25, 26, 27, 28]. Establishing chimerism through donor‐derived hematopoietic stem cell transplantation can lead to central and peripheral tolerance by promoting the deletion or inactivation of donor‐reactive T cells [29, 30, 31]. For instant, in a study indicated that augmenting PD‐L1 expression, either through genetic overexpression or pharmacological intervention, in hematopoietic stem and progenitor cells can effectively reverse autoimmune diabetes by fostering immune tolerance at both central and peripheral levels. When hematopoietic stem and progenitor cells express PD‐L1, they participate in thymic immune re‐education, facilitating the negative selection of autoreactive T cells while simultaneously strengthening regulatory immune mechanisms. This dual action attenuates pathogenic T‐cell responses directed against pancreatic β‐cells, thereby restoring immunological balance and halting autoimmune destruction [27]. Similarly, thymic approaches, such as cotransplantation of donor thymic tissue or intrathymic delivery of donor antigens, can induce central tolerance by reshaping T cell development, allowing the recipient's immune system to accept donor islets without chronic immunosuppression. Preclinical models have shown that intrathymic administration of donor MHC peptides or islets leads to long‐term graft survival and donor‐specific unresponsiveness. Furthermore, recent clinical advances using cultured thymic tissue implantation suggest a feasible translational path [25, 32]. These strategies offer a rational framework for preconditioning *T1D* patients to support durable, immune‐tolerant islet engraftment.

### 1.2 Advancements and Challenges in Transplantation for T1D Treatment

#### 1.2.1 Site Selection and Emerging Solutions

Selecting the optimal transplantation site is crucial for ensuring the survival and functionality of an islet graft, as the site must balance various factors, including immune compatibility, neovascularisation, and procedural feasibility. Various sites, including the liver, kidney, bone marrow, pancreas, and subcutaneous space, have been extensively studied, and their advantages and disadvantages are summarised in Table 1 in site selection tailored to specific transplantation approaches [39, 67, 68, 69, 70, 71]. Despite ongoing research exploring alternative sites, the liver remains the clinically preferred option for islet transplantation, even with its limitations. These challenges, such as the need for lifelong immunosuppression and limited donor availability, underscore the importance of innovative strategies such as cell encapsulation, stem cell therapy, xenotransplantation, and immune material modulation. As these advancements progress, they offer the potential to address current barriers, enhance the effectiveness of islet transplantation, and improve outcomes for patients with diabetes [72, 73, 74].

**TABLE 1:** Comprehensive evaluation of transplantation sites for islet grafting: Vascularisation, immune responses, and surgical considerations.

Transplantation site	Study model & outcome	Advantages	Disadvantages	References
Liver	– Human**/ insulin independence for 11.9 months [33] – Rat**/ NA [34] – Human**/ NA [35] diabetic SCID mice**/insulin independence for 28 days, minimal islets apoptosis [36] – Mice to mice & human**/ 60% & 29% viability [37] – Mice**/ 56% viability [38]	– Highly vascularised – Minimally invasive procedure – Supports vascularisation – Long‐term survival	– Induces instant blood‐mediated inflammatory reaction – Hard to monitor grafts – Portal hypertension, thrombosis, bleeding risks, localised steatosis – Difficult to image or retrieve implanted cells	[33, 34, 35, 36, 37, 38, 39, 40, 41, 42]
Kidney	– Mice & human to mice**/ 100% and 75%–80% viability, respectively [43] – Mice**/ labelling indices ratio: 2.18–2.97 [44]	– Good vascularisation – Easy monitoring and retrieval – Immunologically privileged	– Low oxygen partial pressure – Limited volume for islet grafts	[39, 43, 44, 45]
Bone marrow	– Mice**/ decrease glycaemic index after 1 week and achieving maximum performance after 3–4 week [46] – Human**/ required exogenous insulin injections during follow‐up [47] human**/ not suitable for TX [48] – Mice**/ 100% & 56% survival with & without radiation [49] – Rat**/ normoglycemia up to 4 month [50]	– Protection & vascularisation – Accessibility – Monitoring – Invasiveness	– Promote hyperproliferative diseases due to hyperinsulinemia – High rejection rates due to proximity to immune cells – Oxygen tension – Need for preconditioning (local immune modulation)	[46, 47, 48, 49, 50]
Omentum and peritoneal cavity	– Mice**/ normoglycemia after 13.9 days [51] – Mice**/ normoglycemia after > 60 days [52] – Rat to NOD mice and rabbit, dog, mice to mice**/ increase graft survival in CTLA4Ig group [53] – Tilapia to NOD mice**/ graft rejection after 11 ± 4 days [54]	– Large space – High lymphatic and vascularisation – Good plasticity and capacity	– Invasive surgery – Cytokine‐mediated damage	[51, 52, 53, 54, 55, 56]
Subcutaneous and intramuscular spaces	– Mice**/ diabetes reverse in 91% of cases but normoglycemia for up to 100 days [57] – Mice**/ graft survival until the end of study (100 days) [58] – Human & mice to mice**/ euglycemia for 1 year [59]	– Simple operation – Minimal complications – Easy monitoring and retrieval	– Insufficient oxygen supply – Poor insulin release patterns – Poor glycaemic control	[39, 57, 58, 59, 60]
Pancreas	– Rat & dog**/ normoglycemia for 60 days [61]	– Well‐vascularised microenvironment – Minimal inflammatory response	– High risk of complications – Technically difficult surgery	[61, 62, 63]
Spleen	– Mice**/ viability for 51 ± 2.9 days [64] – Mice**/ normoglycemia for > 100 days [65]	– High vascularisation – Immune tolerance – Better glycaemic control	– Increased risk of haemorrhaging – Possible immune response due to lymphocyte access	[45, 64, 65, 66]

*Note:* * and ** show in vitro and in vivo study, respectively.

#### 1.2.2 Standard Allo‐ and Xeno‐Transplantation Therapy

The Edmonton Protocol, recognized as the pioneering and gold‐standard method for islet transplantation, exemplifies the principles of 3 PM in diabetes care. This protocol involves isolating islets from donor pancreases, processing them with enzymatic digestion, and infusing them into the recipient's portal vein. Initially designed to achieve insulin independence, the protocol's focus has evolved to prioritise the prevention of severe hypoglycemia and restoring patients' hypoglycemia awareness, underscoring the importance of patient‐specific outcomes and risk management. Even in cases where complete insulin independence diminishes over time, the procedure still offers a viable long‐term disease management strategy that significantly improves patient quality of life [75].

From a predictive standpoint, careful patient selection and monitoring enable clinicians to anticipate complication risks and tailor immunosuppressive regimens accordingly. Preventively, islet transplantation reduces the frequency and severity of hypoglycemic episodes, improves glycaemic control, and lowers the progression of microvascular complications, thereby mitigating downstream health risks associated with diabetes [76]. Personalised medicine is evident in the adaptation of immunotherapy to individual immune profiles to minimise rejection and autoimmune recurrence [77]. Despite advances in enzymatic isolation techniques yielding healthier islets, multiple donors are frequently required per transplant, imposing practical and ethical constraints on donor use [13, 78]. This scarcity has catalysed innovative research into alternative islet sources, including xenotransplantation with porcine islets and stem cell differentiation into insulin‐producing β‐cells. Porcine islets, historically significant as therapeutic insulin sources, have demonstrated functional efficacy post‐transplantation, as seen in studies reporting sustained islet‐like cell cluster activity for up to 1 year in *T1D* patients [79]. Nevertheless, xenotransplantation raises predictive concerns regarding immune rejection and the transmission of porcine endogenous retroviruses (PERVs). Cutting‐edge genome editing technologies such as CRISPR‐Cas9 have been harnessed to inactivate PERV sequences in porcine cells, resulting in PERV‐free pigs via somatic cell nuclear transfer, an advancement that addresses viral safety risks and represents a genetic engineering solution for safer transplants [69, 80].

In line with predictive and preventive approaches, biomaterial‐based scaffolds have emerged as a promising strategy to enhance the islet microenvironment post‐transplantation. These scaffolds serve critical functions by anchoring islets at the transplant site, providing mechanical support, and protecting them from hypoxic and inflammatory damage, thereby boosting graft survival and function [81, 82, 83, 84, 85, 86]. Continued research is essential to optimise scaffold composition and integration with host tissue, enabling predictive modelling of transplant outcomes and individualised scaffold design tailored to patient‐specific needs. In conclusion, the future of islet transplantation within the 3 PM framework lies in overcoming donor limitations through innovative cell sourcing and engineering, minimising immunological risks via personalised interventions, and enhancing graft survival with biomaterial technologies. These advances collectively aim to deliver safer, more effective, and patient‐tailored treatments that significantly improve the quality of life and revolutionise diabetes management.

#### 1.2.3 Nano‐, Micro‐ and Macro‐Encapsulation

Isolated islets intended for transplantation inherently lack their native vascular networks and specialised extracellular matrix (ECM), making them heavily reliant on host‐derived nutritional and physical support for successful engraftment and long‐term function. Following transplantation, the formation of new blood vessels around and within the graft is critical to meet the metabolic demands of these cells, representing a key predictive factor influencing transplant success [45, 87, 88, 89]. Within the framework of 3 PM, revascularisation dynamics, oxygen gradients, and inflammatory responses should be recognized as early predictive biomarkers that inform individualised therapeutic optimization [90].

Encapsulation of islets within biocompatible polymers offers an innovative preventive strategy to shield transplanted cells from immune‐mediated destruction without systemic immunosuppression, thus reducing associated risks. This approach facilitates selective permeability, allowing the exchange of essential nutrients, metabolites, and insulin while protecting islets from host immune cells [91, 92, 93, 94]. To further enhance graft performance, integrating bioenergetic and mitochondrial health indicators into the evaluation process can provide predictive insights into cell vitality, enabling timely optimization of scaffold architecture or co‐transplantation components, such as antioxidant‐enriched biomaterials and mitochondrial cofactors [95]. Encapsulation techniques can be implemented across different scales, macro, micro, and nano, each presenting unique benefits and limitations that need to be considered in therapeutic design (Table 2) [133, 134, 135, 136, 137, 138, 139, 140, 141].

**TABLE 2:** Advanced encapsulation strategies for islet cell transplantation: Innovations in macro‐, micro‐, and nanoencapsulation techniques to enhance diabetes treatment outcomes.

Category	Description	Materials/techniques	Study model & outcome	Advantages	Challenges	Examples/applications
Macro‐encapsulation	Encapsulates a substantial mass of cells within a device. Provides non‐invasive benefits and greater availability compared to whole‐organ transplantation. Common transplantation sites include intraperitoneal, omental, and subcutaneous spaces.	– Polylactic acid (PLA) – Poly(ethersulfone) (PES)/polyvinyl pyrrolidone (PVP) – Silicon nanopore membrane (SNM) – Electrospun nanofibers – 3D printing with biocompatible materials – Polyethylene glycol (PEG) – Alginate – Hyaluronic acid (HA) – Poly(lactide‐co‐glycolide) (PLG) – Fibrin‐based gel – Poly(lactic‐co‐glycolic) acid (PLGA) – Polydimethylsiloxane (PDMS) + fibrin + platelet‐derived growth factor (PDGF) – Gelatin‐collagen matrixes – Alginate‐based bio‐ink – Alginate	– MIN6‐B1 mouse, human islets*/ Functional islets for 7 days [96] – Mouse islets*/ response to glycaemic change for 6 hr [97] – hES‐βC to mice*, **/ decrease FBR [98] – Mouse pancreas**/ decrease viability in high dose of FTY720 [99] – Rat to mice and human to SCID Beige mice**/ viability for 3 and 4 months, respectively [100] – Rat*/ viability 75%–85% after 6 day [101] – Rat**/ functional for > 150 days [102] – Rat**/ euglycemia > 7 months and viability > 90% [103] – Rat**/ improve viability [104] – Mice**/ viability 85%–90% after 1–3 days [105] – Mice**/ viability < 60% but MRT = 84 days in group without aprotimin [106] – Mice**/ improve viability [107] – Mice**/ delay engraftment, viability 33 days [108] – INS1 cells*/ improve viability [109] – Rat islets*, **/ improve response for insulin secretion up to 240% and euglycemia for > 120 days [110] – Rat**/ normoglycemia for 210 days, long term survival [111]	– Non‐invasive – More islets transplanted compared to micro/nano methods – Provides mechanical support	– Potential for fibrosis – Vascularisation around the device – Long‐term stability issues	– PES/PVP membranes for islets: High stability, biocompatibility, blocking immune cells [96] – SNM: Selective permeability, protection against immune components [97] – Zinc oxide nanorods/PCL: Protect cells from the host immune system while allowing ideal glucose and insulin exchange [98] – Electrospun nanofibers: Barrier to inflammatory cells, local release of FTY720 for immunomodulatory effects [99] – TRAFFIC: Free of fibrosis, most of the islets remaining viable [100] – PEG‐based hydrogel microdevice functionalised with VEGF and RGD: Enhanced local vascularisation, improved oxygen and nutrient delivery, desirable insulin responsiveness, and islet immunoisolation [101] – NICHE: Vascularisation, localised immunosuppression [102] – Alginate slab + O2 gas chamber: Provided with exogenous oxygen for encapsulated islets [103] – Oxysite: Generate sufficient local oxygen, enhanced vasculature formation [104] – Microneedle patch: Maintenance of blood glucose levels in the normal range for about 6 h [105] – Fibrin gel with VEGF: Improve islets function [106] – Fibrin based gel with recombinant human fibronectin fragment containing integrin and binding domains for VEGF and PDGF: Enhances the vessel branching and efficiency of engraftment [107] – PDMS + fibrin + PDGF: Enhance islet engraftment [107] – PLG with IL‐33 modulates innate immune cells and extendsislets' survival [108] – In fabricated stiff multilayer GelMA matrixes by digital light processing, pancreatic *β* cells spontaneously formed aggregates [109]. – The enhancement of insulin secretion function by 240% facilitates a rapid recovery of blood glucose levels in less than 1 day, with sustained stability exceeding 120 days [110]. – The alginate macrocapsule‐device maintained normoglycemia for 210 days post‐transplantation [111]
Micro‐encapsulation	Involves enclosing one or a few cells within semipermeable microcapsules, typically less than 1 mm in diameter. This strategy aims to immunoisolate transplanted cells using various biomaterials.	– Alginate – Poly L‐lysine – Biotinylated PEG microgels	– Rat to mice**/ normoglycemia up to 30 days [112] – Murine**/ increase viability and improve glycaemic index [113] – Non‐human primates**/ After 6 months, very few functional islets remained [114] – Mice**/ proper glycaemic index for > 150 days, 100% viability in CXCL12 (2ug/ml) group [115] – Mice**/ control glycaemic for 80 day but graft reject after 19 days [116] – BalB/C murine and porcine to C57BL/6 mice **/ normoglycemia > 100 days in CXCL12 (1ug/ml) group [117] – Non‐human primates**/ graft survival > 6 month [118]	– Immunoisolation – Reduced need for immunosuppressants – Biocompatibility – Enhanced nutrient/waste exchange	– Formation of fibrotic cell layers – Long‐term stability – Need for localised delivery of agents	– Alginate microcapsules with dexamethasone and curcumin: Reduced foreign body response (FBR), improved long‐term release of insulin [112] – Alginate hydrogel with dexamethasone‐loaded PLGA microspheres: Reduced inflammation, enhanced islet viability [113] – Co‐encapsulation with CXCL12: Recruitment of T*reg* cells, reduced FBR [114, 115] – PLG/poly(vinyl alcohol (PVA)) microspheres with TGF‐β: decrease inflammation and enhance islets function [116] – Islet microencapsulation in alginate with CXCL12: Local immune‐isolation and long term survival and function [117] – Streptavidin‐FasL‐PEG microgels, localised tolerance in islet allograft [118]
Nano‐encapsulation	Encapsulates individual cells in a nanoscale shell, providing improved immuno‐isolation and facilitating the diffusion of oxygen, nutrients, and waste. Techniques include PEGylation and layer‐by‐layer coating.	– PEG – Layer‐by‐layer coating – Multilayer nanocoating – Hyperbranched PEG/heparin – Zinc oxide nanorods – Polycaprolactone – Polydimethylsiloxane – Polycaprolactone (PCL)/heparin – liposomes	– Non‐human primates**/ graft survival for weeks to months [119] – Mice**/ diabetes improvement comparable to control group [120] – Rat**/ viability for 15 ± 2.1 days [121] – Non‐human primates**/ survival time = 108 days [122] – Non‐human primates to mice**/ euglycemia after 2–3 week [123] – Rodent**/ viability up to 100 days in CSA group [124] – Rat (SD) to inbred F344 rat**/ viability > 100 days [125] – Murine**/ normoglycemia > 100 days and 78% of the graft exhibiting euglycemia [126] – Non‐human primates to mice**/ 100% survival for 150 days [127] – Mice**/ be functional during a 1‐month study period and normoglycemia up to 45 days [128] – Human islets*, human to mice**/ viability > 90% [129] – Mice*/after 7 days have functional response [130] – Rat to nude mice**/ normoglycemia for 14 days in 80% of cases with aFGF 1ug [131]	– Enhanced biological responsiveness – Facilitated diffusion – Reduced immune response – Improved release of biomolecules	– Technical complexity – Consistency in coating – Long‐term effectiveness	– PEGylated islets: Prolonged survival, function, immune rejection prevention [119, 120, 121, 132] – PEG + heparin nano‐shielded islets: Immune protection [122] – Hyperbranched PEG/heparin islets nanoencapsulation: Immune protection and reducing proinflammatory cytokine generation [123] – PEGylated islets with cyclosporine A (and heme oxygenase‐1): Immunologically protect the islets [124, 125] – PEGylated islets with LFA‐1 antibody: Immunoprotective effect [126] – Layer‐by‐layer nano‐shielding with tacrolimus, sirolimus, anti‐LFA‐1: Enhanced insulin release, reduced immune cell infiltration [127] – Multilayer coatings with chitosan, alginate: Enhanced islet function, protection against cytokines [128] – Layer‐by‐layer nano‐encapsulation of chitosan and polystyrene sulfonate sodium salts: Enhanced function of islets and protected the cells against the inflammatory cytokine damage [129] – PCL scaffold with heparinised surface containing VEGF: Enhances angiogenesis and islet function [130] – aFGF‐liposomes: Improve vascularisation and the immediate restoration of blood glucose levels in a short time [131]

*Note:* * and ** show in vitro and in vivo study, respectively.

Macroencapsulation involves enclosing islets within a large, semi‐permeable device that allows for straightforward retrieval, if necessary, a feature aligning with safety considerations. However, their considerable size impairs the efficient diffusion of oxygen and nutrients to encapsulated cells, often jeopardising cell viability. Moreover, macro devices tend to provoke a foreign body reaction (FBR), triggering fibrotic tissue formation that further hinders nutrient exchange and graft function, thus posing a significant preventive challenge for long‐term success [142, 143, 144]. Its large diffusion distances and strong FBR underline the importance of monitoring systemic inflammation as a biomarker for fibrotic overgrowth and graft failure. Predictive modelling using AI‐driven image and biomarker analysis could support early detection and intervention [145, 146].

Microencapsulation provides a more favourable microenvironment by encapsulating individual or small clusters of islets within semipermeable membranes. This smaller scale enhances cell viability and functionality, and the minimally invasive nature of microcapsules supports personalised implantation strategies tailored to patient needs [147]. To further improve post‐transplant success, integrating digital health technologies, such as continuous glucose monitoring combined with inflammatory biomarker profiling, can generate predictive datasets that guide adaptive management and early detection of graft dysfunction [20]. Nevertheless, the clinical translation of microencapsulation still faces critical challenges, including difficulties in localising grafts after transplantation, which complicates effective monitoring and timely interventions. In addition, precise control over membrane pore size and thickness is essential to balance nutrient and oxygen diffusion with immune protection; however, current fabrication techniques have yet to achieve this level of uniformity and reproducibility [148, 149, 150].

Nanoencapsulation utilises nanoscale materials to coat or encapsulate islets, significantly reducing thrombosis risk due to minimal interference with blood components, a predictive advantage in anticipating vascular complications. Nonetheless, nanoparticle instability raises concern about premature degradation and unintended release of encapsulated cells or agents. Moreover, the immune system's recognition of nanoparticles can provoke adverse reactions, which restrict their clinical use unless personalised immunomodulatory approaches, potentially guided by AI‐driven material optimization, are employed to strike an optimal balance among stability, biodegradation, and immune compatibility [151, 152, 153].

A comparative evaluation of encapsulation methods indicates that no single technique provides a universally superior outcome across all dimensions of islet transplantation; however, microencapsulation currently presents the most balanced and 3 PM‐consistent approach [93, 154, 155]. It combines effective immunoprotection, adequate nutrients and oxygen diffusion, and adaptability to individualised therapeutic needs while minimising the FBR [154, 155, 156]. Within the 3 PM framework, microencapsulation embodies the personalised component by enabling design modifications, such as capsule size, permeability, and immune barrier strength, according to each patient's inflammatory and metabolic phenotype. From a predictive perspective, early biomarkers such as revascularisation rate, oxygen gradients, inflammatory mediators, and mitochondrial activity can serve as reliable indicators of graft viability. Integrating AI‐based modelling and digital monitoring systems that combine these molecular and physiological parameters can help forecast graft outcomes and guide timely clinical interventions [154, 155, 157, 158, 159, 160, 161]. In parallel, preventive measures, through localised immunomodulation, antioxidant loading, or bioenergetic support, address systemic inflammation and mitochondrial stress before irreversible graft damage occurs [156, 161, 162]. Future progress should prioritise the creation of multifunctional biomaterials that couple mechanical stability with biological intelligence, such as embedded biosensors for continuous metabolic monitoring or materials that dynamically modulate the immune microenvironment. Incorporating these features transforms encapsulation systems into smart therapeutic platforms capable of real‐time adaptation to patient‐specific needs [93, 154, 163].

**FIGURE 1:**
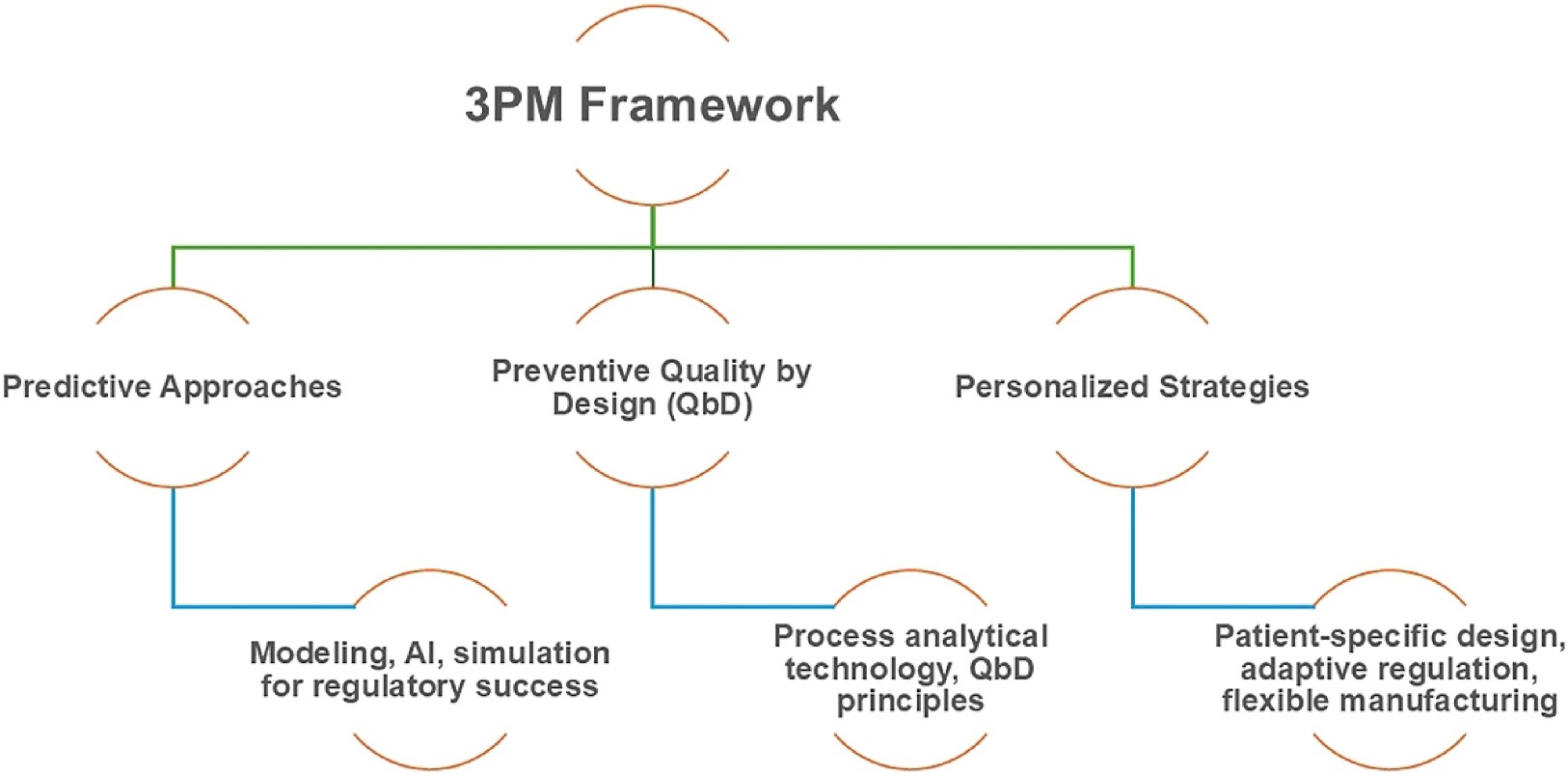
Integration of predictive, preventive, and personalised medicine (3 PM) principles into regulatory and manufacturing strategies for encapsulated islet therapies.

However, realizing this vision requires addressing the substantial regulatory and manufacturing hurdles that currently impede the clinical translation of biomaterial‐based islet encapsulation therapies. These challenges encompass complex regulatory classification issues, standardisation of manufacturing processes, quality control requirements, biocompatibility assessment, and economic considerations [155, 163, 164, 165]. Within the 3 PM framework, addressing these obstacles requires predictive regulatory strategies, preventive quality control measures, and personalised approaches to material selection and manufacturing protocols (Figure 1). The current regulatory landscape lacks harmonised guidelines specifically tailored to encapsulation technologies, while manufacturing processes struggle with reproducibility, scalability, and standardisation, critical factors that determine both regulatory approval and commercial viability [155, 161, 163, 166].

Despite these challenges, the convergence of advanced biomaterial science, predictive analytics, and personalised medicine principles offers a clear pathway forward. By merging predictive diagnostics, preventive immunomodulation, and personalised material design, islet encapsulation technologies can advance beyond reactive transplantation practices towards a holistic, multi‐modal strategy aligned with 3 PM principles. This paradigm shift, supported by international harmonisation efforts and innovative manufacturing approaches, is expected to improve graft longevity, patient safety, and clinical accessibility, ultimately fulfiling the vision for the 3 PM approach to *T1D* management [157, 160, 161, 165].

#### 1.2.4 3D Printing

Three‐dimensional (3D) bioprinting represents a cutting‐edge fabrication technology that merges living cells with biocompatible hydrogels to generate complex 3D biological structures. Essentially, it functions analogously to print living tissues and organs, constituting an advanced subset of additive manufacturing tailored for biomedical applications [167, 168, 169]. The primary impetus behind 3D bioprinting lies in addressing the pressing need for biological constructs to regenerate damaged tissues and organs, ultimately aiming to develop functional substitutes that alleviate dependency on donor tissues and reduce the use of animal models in research and drug development, aligning with personalised and preventive therapeutic strategies.

Unlike conventional 3D printing, 3D bioprinting presents unique challenges because it involves encapsulating sensitive living cells within bio‐inks that must preserve cell viability while avoiding cytotoxicity from materials and environmentally stressful conditions during the printing process [170, 171]. The overarching goal is to engineer living constructs that restore physiological tissue and organ functions in a patient‐specific manner, aligning with the principles of personalised medicine. This technology enables the creation of phenotypically tailored constructs reflecting each patient's molecular, metabolic, and immunological profile, an essential factor for optimal engraftment and long‐term tolerance. Also, by enabling micrometre‐level precision in depositing cell‐laden bio‐inks, 3D bioprinting overcomes critical limitations of traditional scaffold‐based tissue engineering, such as poor spatial control and inconsistent cell distribution [172, 173]. Within a 3 PM context, this challenge translates to preventive control of cellular stress, mitochondrial dysfunction, and systemic inflammation, factors known to compromise graft survival and patient outcomes [95].

Notably, this approach has been applied to islet transplantation, where beta cells and islets have been successfully bioprinted within hydrogel matrices. Integrating mitochondrial stress assessment and AI‐driven image or secretion analyses can identify optimal conditions for maintaining islet function during and after bioprinting. Although these constructs have demonstrated cell viability, functional assays reveal reduced insulin secretion in response to glucose, highlighting a current preventive challenge to fully replicating native islet physiology post‐bioprinting. Nevertheless, bio‐printed scaffolds offer important advantages over simple hydrogel encapsulation, including increased surface area‐to‐volume ratios and enhanced potential for vascular ingrowth. This vascularisation is critical for improving oxygen and nutrient delivery to transplanted cells, directly addressing predictive factors influencing graft survival [174].

As the biodegradable scaffold materials gradually degrade in vivo, engrafted islets and surrounding host tissues deposit ECM proteins, thereby reconstructing the native microenvironment essential for islet survival and function, further emphasising the importance of preventive strategies tailored to optimise engraftment conditions [172, 174]. Table 3 summarises studies employing 3D bioprinting and various biomaterials to fabricate islet cell scaffolds, highlighting ongoing efforts towards personalised transplantation strategies. Novel bioinks incorporating immunomodulatory or antioxidant agents, as well as co‐transplant support cells such as MSCs, exemplify 3 PM principles by enabling localised, patient‐specific immune regulation instead of systemic immunosuppression. These strategies reflect the broader transition from reactive care to predictive and preventive medicine, emphasising immune balance over global suppression [201, 202]. Translationally, 3D bioprinting can serve as a digital twin platform for predictive simulation, combining AI‐driven design, multi‐modal diagnostics (imaging, omics, and digital biomarkers), and real‐time health monitoring to forecast graft performance. Integration of in silico modelling with patient phenotyping data allows the prediction of bioenergetic and inflammatory responses to scaffold designs prior to clinical application, thus advancing the core aims of 3 PM [203, 204, 205].

**TABLE 3:** Comprehensive overview of scaffold materials, cell types, and outcomes in islet cell transplantation and bioprinting research (2011–2025): Innovations in diabetes treatment.

Studies	Material/cells	Studies model	Result
1	Poly(lactic‐co‐glycolic) acid (PLGA), human islets	*Human	Printed scaffold‐induced insulin secretion and endocrine gene expression compared to gels without a 3D scaffold [175].
2	Alginate, gelatin, hyaluronic acid, matrigel, rat INS1E β‐cells, mouse islets, human islets	*Porcine	Reduced nutrient diffusion through the gel is thought to hinder insulin secretion [176].
3	Alginate, matrigel, gelatin, HÀ, INS1E β ‐cell	*Human and mouse	The high density of hydrogel decreased nutrient diffusion within the gel and might hamper insulin secretion [173].
4	Alginate, mouse βTC‐3 cells/rat dermal fibroblast cells	*Mice	The formation of scaffold‐free cell aggregates in the form of cylindrical strands could be fused together and maintain the viability and functionality of cells that secrete insulin in tissue strands [177].
5	Polylactic acid (PLA), differentiated human ESCs (SC‐β cells)	**Mice	Serum insulin detection and C‐peptide staining after 12‐week implantation in the subcutaneous space of non‐diabetic SCID beige mice [178].
6	PLA, human islets	**Human	VEGF‐loaded scaffolds in the subcutaneous space of nude mice and insulin detection for up to 22 weeks after transcutaneous refilling [179].
7	PLA/Fibrinogen, human stem cell‐derived β‐like cells	*Mice	Twelve weeks after implantation in the subcutaneous space of non‐diabetic SCID mice, serum insulin detection and C‐peptide staining were positive [180].
8	Alginate, mouse βTC‐3 cells	*Mice	Maintenance of viability and detection of insulin secretion in 9tissue strands that could be fused [181].
9	Medical grade polylactic acid, human islets	**Mice	Scaffolds were implanted 4 weeks before the injection of human islets into the subcutaneous site of the nude non‐diabetic mice. Followed by 2 doses of human islet (2000 IEQ, and 2000 IEQ, second dose 12 weeks later). The islets stayed viable as the scaffolds protected them from hypoxia throughout the study [182].
10	Alginate/methylcellulose, rat islets	*Rat	The islets survived and insulin/glucagon was positive, but after glucose stimulation, increased apoptotic cells and reduced insulin secretion in printed islets versus free islets were seen [183].
11	Alginate/gelatin methacryloyl (GelMA), mouse islets, EPCs, T*reg*	*Mice	Printing the core‐shell construct, which keeps the mouse islets in the core and EPCs and T*regs* in the shell as an immune protective layer, reduced insulin secretion after glucose stimulations [184, 185].
12	PLGA with salt particles, human islets	*Human	Increased insulin response and endocrine gene expression in printed scaffold compared to gels without 3D scaffold [186].
13	Decellularized extracellular matrix (dECM), rat islets, human islets, differentiated hiPSCs, INS‐1 cells	*Rat, human	Decellularized pancreatic tissue printed in gel form allows for similar viability after 5 days to non‐printed gel [187].
14	Alginate (Alg) + polyethylene oxide (PEO), MIN6 cell	*Human	Live/dead assay reveals that bio‐inks made of Alg4.0 and Alg1.5PEO8.0100k, respectively, exhibit a viability of 95.3 ± 1.5% and 90.4 ± 0.5%. DASP (digital assembly of spherical particles) encapsulated human islets exhibit a glucose stimulation index of 2 compared to the control group (around 1) [188].
15	Alginate + dECM, in vitro: Porcine pancreatic islets	*Porcine	Insulin secretion upon glucose stimulation and the development of vessel‐like structures composed of CD31‐positive cells validated the biomimetic potential of the two formulated bioinks [189]
16	Polycaprolactone (PCL)/dECM, human pluripotent stem cells (hPSC)/MIN6‐m9 cells	*Human	The insulin‐producing β‐cells printed in porous hybrid scaffold systems were better than the nonporous‐type, 3D‐bioprinted pancreatic islet‐like aggregates in terms of viability and function, and induced structural maturation and functional enhancement, as well as polarization of M1 macrophage [190].
17	Alginate microcapsule, neonatal porcine islet‐like cell clusters (NICC)	*Porcine	The number of insulin‐positive cells is comparable between the groups and over time. The amount of insulin release increases over time and is released in response to glucose stimulation over 4 weeks [191].
18	PCL/GelXA LamininK‐411 (alginate, GelMA, laminin), INS1, HUVEC	*Rat	Heparin‐functionalised PCL increased VEGF expression and cell adhesion, and commercial mixed hydrogel induced β‐cell proliferation and insulin secretion [192].
19	dECM, pig	*,**Pig	Stable blood flow within a transplanted bionic organ was observed for 2 weeks' post‐transplant. Histopathological examination showed the presence of CD31/PECAM‐1 [193].
20	dECM, gelatin methacrylate (GelMA) and methacrylic hyaluronic acid (HAMA), porcine islet	**Murine	During the 21‐day observation, it was shown that the cells encapsulated by the inkjet method show practically 100% viability. On the 14th day of the experiment, cells suspended in bioink with dECM showed a definite superiority in response to the administered glucose. Compared to the control group, the increase was over 50% [194].
21	Alginate‐ nanofibrillated cellulose, mice	*,**Human, mice	Diabetic mice transplanted with islet‐adipose‐derived stromal cells (ASC) scaffolds reach normoglycemia 7 days' post‐transplantation with no significant difference between this group and the group that received islets under the kidney capsules. In addition, animals transplanted with islet‐ASC scaffolds stay normoglycemic and show elevated levels of C‐peptide compared to mice transplanted with islet‐only scaffolds [195].
22	dECM hydrogel, MIN‐6 pancreatic beta cells	*,**Mice	A 3D‐printed microdevice with vascularised β‐like cells achieved long‐term glycaemic control in diabetic mice, providing a safe and potentially transformative therapy for type 1 diabetes. In this approach, pancreatic islet cells were protected against immune cell infiltration mediated by inflammatory factors and prevented insulitis [196].
23	VEGF‐calcium peroxide biomaterial scaffold (l‐lactide and D,l‐lactide)	*,**Rat	This scaffold normalised blood glucose levels in diabetic rats and exhibits excellent biocompatibility, minimising adverse reactions [197].
24	GelMA hydrogel, UiPSCs‐derived β‐like cells and islet cells	*,**Human, mice	A 3D‐printed and retrievable microdevice containing vascularised β‐like cells achieved long‐term glycaemic control in a subset of diabetic mice, offering a promising therapeutic strategy for T1D patients [198].
25	Porcine dECM, stem cell‐derived islets	*Porcine	A bioprinted pancreatic niche improves the structural and functional maturity of stem cell‐derived islets [199].
26	Alginate core‐shell fibres, MIN6, human islets	*Mice, human	Islets demonstrating glucose‐responsive insulin secretion [200].

*Note:* * and ** show in vitro and in vivo study, respectively.

In conclusion, 3D bioprinting represents not only a manufacturing breakthrough but also a cornerstone of 3 PM‐oriented regenerative medicine. It supports (i) predictive modelling of graft performance via AI and bioenergetic profiling, (ii) preventive control of immune, oxidative, and mitochondrial stress through optimised bioinks and localised immunomodulation, and (iii) personalised scaffold designs tailored to individual immune–metabolic profiles. Together, these advances drive the transition from reactive transplantation towards integrative and 3 PM therapies for diabetes and related disorders.

#### 1.2.5 Immunomodulatory Strategies and Genetic Engineering of Islet Cells: Enhancing Survival and Function

The persistent reliance on chronic systemic immunosuppression, the current mainstay of allotransplantation, highlights an urgent need for more innovative and targeted therapeutic strategies. Although these regimens have markedly improved short‐term graft survival and can enable transient insulin independence following islet transplantation, their long‐term use carries substantial risks. Patients face heightened susceptibility to infections and malignancies, drug‐induced organ toxicity, and a progressive decline in graft function, with insulin independence typically falling to approximately 25% at 5 years [206, 207, 208]. These challenges are further compounded by donor organ shortages, often necessitating multiple donors per recipient. Collectively, these limitations underscore the difficulty of predicting individual risk profiles and emphasise the necessity for precision immune modulation strategies that promote graft tolerance while minimising off‐target effects [206, 208, 209].

In response, emerging pharmacological approaches aim to reshape the graft microenvironment rather than broadly suppress systemic immunity. For instance, human embryonic stem cell‐derived islets (SC‐ islets) have been genetically engineered to secrete tolerogenic cytokines—such as IL‐10, TGF‐β, and modified IL‐2, creating a localised immunoregulatory niche. In preclinical models, these modified islets recruit T*regs*, resist both xenogeneic and allogeneic rejection, and restore normoglycemia in non‐obese diabetic (NOD) mice for up to 8 weeks without encapsulation or systemic immunosuppression [209, 210]. Complementary pharmacotherapies, including combinations of 1α,25‐dihydroxyvitamin D3 and mycophenolate mofetil, further promote tolerance by inducing dendritic cells with a tolerogenic phenotype, characterised by downregulated costimulatory molecules (CD40, CD80, CD86), suppressed proinflammatory cytokine production (IL‐12, IFN‐γ), and expansion of CD4+ CD25+ CD152+ T*regs*, enabling adoptive transfer of islet allograft tolerance [211].

These interventions operate by attenuating inflammatory signalling and immune‐mediated stress within the graft, thereby preserving β‐cell metabolic fitness, insulin secretory capacity, and mitochondrial integrity, key determinants of long‐term islet survival. By modulating immune‐metabolic crosstalk and cellular stress‐response pathways, such strategies reduce islet vulnerability to alloimmune injury while maintaining intrinsic function. Critically, this localised approach avoids compromising systemic immune competence, positioning targeted pharmacologic modulation as a promising adjunct to conventional transplantation protocols [209, 211].

Parallel advances in genetic engineering offer another path towards immunosuppression‐free transplantation. Clinical‐stage applications of CRISPR‐Cas9/12b and lentiviral technologies have enabled precise modification of allogeneic donor islets to evade immune recognition. Early human trials involving forearm‐transplanted, gene‐edited islets have demonstrated stable C‐peptide production and glucose‐responsive insulin secretion at 12 weeks' post‐transplantation—without immunosuppression or serious adverse events. Strategies targeting HLA class I/II molecules, upregulating immune checkpoint ligands such as PD‐L1, and co‐expressing tolerogenic cytokines further enhance the immune‐evasive properties of SC‐islets, addressing scalability challenges in β‐cell replacement therapy [206, 209, 212, 213, 214, 215].

Importantly, non‐genetic strategies provide complementary avenues that avoid potential genomic risks such as insertional mutagenesis or oncogenic transformation. Human SC‐islets exhibit intrinsic resilience to inflammatory stress, maintaining glucose responsiveness and endocrine identity through cell‐autonomous signalling pathways that limit cytotoxic T cell–mediated destruction without altering antigen presentation [209, 216, 217]. This resilience is further amplified by MSCs, which exert paracrine immunomodulatory and trophic effects by suppressing effector immune cells while promoting β‐cell survival and tissue repair. Early‐phase trials in both *T1D* and *T2D* have shown promising results with MSC co‐transplantation [209, 216, 217, 218, 219].

**FIGURE 2:**
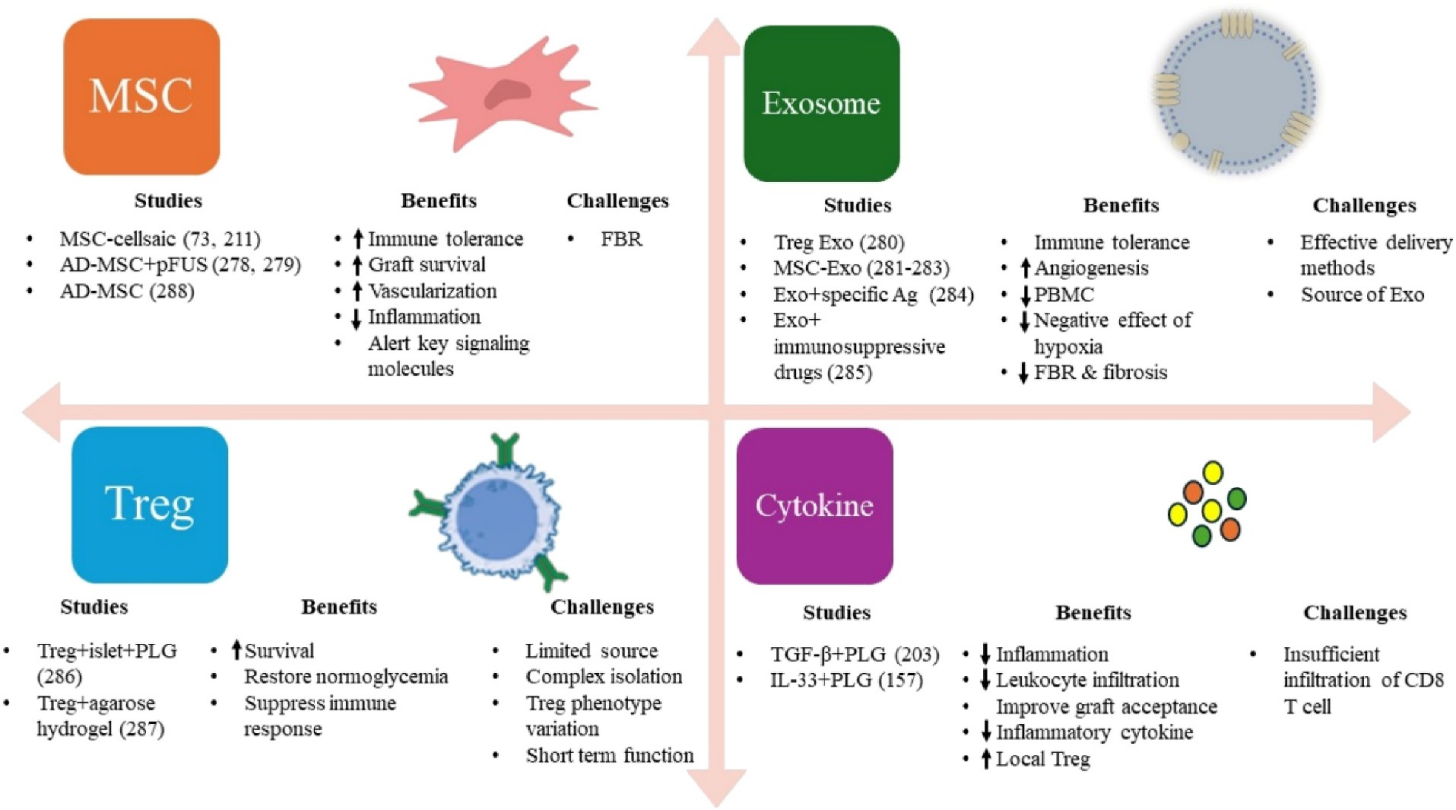
Comprehensive strategies for immunomodulation in islet cell transplantationexplore the role of MSCs, exosomes, engineered T*regs*, and regulatory cytokine co‐delivery to enhance graft survival and function [108, 116, 142, 220, 221, 222, 223, 224, 225, 226, 227, 228, 229, 230, 231].

Aligned with the principles of 3 PM, next‐generation biomaterial platforms integrate these cellular and molecular tools for localised, spatiotemporally controlled delivery. Scaffolds functionalised with MSCs, engineered T*regs*, exosomes, or cytokines (e.g., IL‐10, TGF‐β, modified IL‐2) enable site‐specific immunomodulation, circumventing the pitfalls of systemic drug exposure (Figure 2) [209, 219, 232, 233, 234]. Extracellular matrix based systems further support graft integration by facilitating vascularisation, stem cell homing, and neural regeneration while enabling sustained release of therapeutic agents with minimal toxicity [209, 211, 216, 217, 218, 232, 234].

Realizing the full potential of these strategies will require multifunctional, biodegradable biomaterials tailored to individual immune phenotypes. Future integration with AI‐driven monitoring and multi‐omics profiling promises to enable dynamic, predictive therapy adjustment [232, 234]. Together, this integrated continuum, spanning pharmacologic microenvironment engineering, genetic and non‐genetic immune modulation, and smart biomaterial platforms, marks a paradigm shift in allotransplantation: from reactive, broad immunosuppression towards proactive, patient‐tailored strategies that foster durable graft acceptance with minimal long‐term therapeutic burden.

### 1.3 Optimising Biomaterial Integration: Navigating Immune Responses and Enhancing Tolerance

#### 1.3.1 Foreign Body Reactions to Biomaterials: Immune Response and Biocompatibility Strategies

**FIGURE 3:**
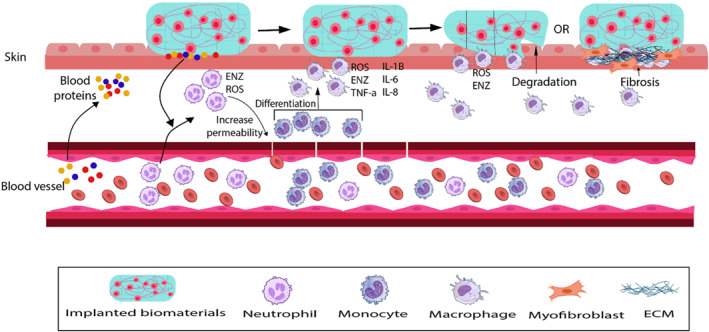
Host immune response to implanted biomaterials: pathways to degradation or fibrosis formation. Schematic illustration of the mechanisms involved in the foreign body reaction to implanted biomaterials. The implanted biomaterials either degrade over time or become coated with fibrotic tissue.

Directly following implantation, the biomaterial surface rapidly becomes coated with a variety of host proteins, initiating the acute inflammatory and subsequent chronic fibrotic stages of the FBR. This response arises from a physicochemical mismatch between the implanted device and native host tissue, posing a major predictive challenge in anticipating device‐host interactions and implant outcomes (Figure 3) [235, 236, 237, 238, 239, 240, 241].

The acute phase begins with tissue injury and absorption of blood proteins such as albumin and fibrinogen onto the implant surface, forming a provisional matrix that triggers immune cell recruitment. Neutrophils are the first responders, releasing reactive oxygen species (ROS) and proteolytic enzymes that amplify inflammation and increase vascular permeability. Monocytes differentiate into macrophages that secrete pro‐inflammatory cytokines, including tumour necrosis factor‐alpha (TNF‐α), interleukin (IL)‐1β, IL‐6, and IL‐8, thus sustaining inflammation locally. Macrophage adhesion to the implant is mediated by integrins like αMβ2 binding fibrinogen on the surface. When the implant exceeds phagocytic size, macrophages engage in frustrated phagocytosis, releasing enzymes and ROS that degrade the implant surface and may cause toxic leakage. If degradation proceeds successfully, inflammation resolves; if not, persistent macrophage presence leads to further recruitment and formation of an isolated macrophage layer, marking the transition to chronic FBR [242, 243, 244].

In the chronic phase, molecular signalling shifts from inflammation to fibrosis, representing a preventive challenge where long‐term implant function can be compromised. Macrophages transition from a pro‐inflammatory M1 phenotype to an anti‐inflammatory M2 phenotype by releasing IL‐10 and transforming growth factor‐beta (TGF‐β). These cytokines suppress inflammation and recruit fibroblasts, which, upon activation by TGF‐β, transdifferentiate into myofibroblasts that deposit ECM proteins to encapsulate the implant in fibrotic tissue. Platelet‐derived growth factor (PDGF) promotes myofibroblast proliferation, further thickening the capsule. Macrophages also fuse into foreign body giant cells on the implant surface, primarily contributing through phagocytosis without driving novel signalling. As the fibrotic capsule matures, nutrient limitations induce the release of vascular endothelial growth factor and PDGF, stimulating angiogenesis to sustain the fibrotic tissue, although this fibrotic barrier impedes effective signal transduction and drug delivery, ultimately impairing implant functionality [244, 245, 246, 247].

These molecular and cellular mechanisms illustrate why FBR remains a persistent obstacle, necessitating implant designs that are predictive of fibrotic potential and preventive against excessive inflammatory and fibrotic responses. Key strategies to modulate FBR and promote graft efficacy include: (1) optimising material chemical composition and mechanical properties to reduce immunogenicity and promote a tolerogenic response; (2) employing biocompatible materials such as polyethylene glycol or zwitterionic hydrogels to minimise protein adsorption and immune activation; (3) leveraging biodegradable or shape‐modifiable materials that elicit favourable wound healing; (4) utilising living‐donor transplants which show lower immune reactivity compared to deceased donor grafts; (5) selecting well‐vascularised implantation sites to improve nutrient delivery and integration; and (6) enhancing local angiogenesis to support implant survival and function. Intriguingly, certain controlled inflammatory responses can be beneficial by promoting neovascularisation, thus supporting graft viability, exemplifying the need for personalised modulation of the local immune environment [235, 248, 249, 250].

In summary, the FBR poses a complex challenge in biomedical implantology, requiring predictive insight into immune‐material interactions, strategies to reduce fibrosis and inflammation, and approaches tailored to patient‐specific biology. By integrating immunology, materials science, and tissue engineering, next‐generation implants are being designed to better harmonise with host tissues. Personalised and regenerative strategies hold promise to optimise clinical outcomes and enhance patients' quality of life.

#### 1.3.2 Tolerance Induction, Research, and Challenges Ahead

As highlighted above, innovative strategies in biomaterials and immunomodulation are being increasingly integrated into islet transplantation research, particularly to optimise therapeutic outcomes for *T1D* within the framework of 3 PM. Predictively, the identification of patient‐specific immune profiles, fibrotic risk signatures, and tolerance biomarkers guide the rational design of biomaterial scaffolds and drug delivery systems. Preventively, biomaterials are engineered not only to mitigate fibrosis but also to minimise early inflammatory responses, thereby reducing the likelihood of graft loss. Personalised approaches rely on tailoring immunomodulatory regimens to the patient's unique immune landscape, maximising islet survival and long‐term graft function.

Recent advances in biomaterial‐based delivery systems illustrate this shift. Polymers such as PLA and PLGA remain highly attractive due to their biocompatibility and versatile encapsulation properties, allowing precise release of anti‐inflammatory, immunosuppressive, or tolerogenic agents [251, 252, 253, 254, 255]. Lipid nanoparticles, particularly those enriched with phosphatidylserine, demonstrate intrinsic tolerogenic effects and align with preventive strategies by dampening innate immune activation [256, 257, 258, 259, 260]. Similarly, inorganic nanoparticles such as gold and silica offer non‐toxic, immunomodulatory properties capable of fine‐tuning innate immune responses [261, 262].

**FIGURE 4:**
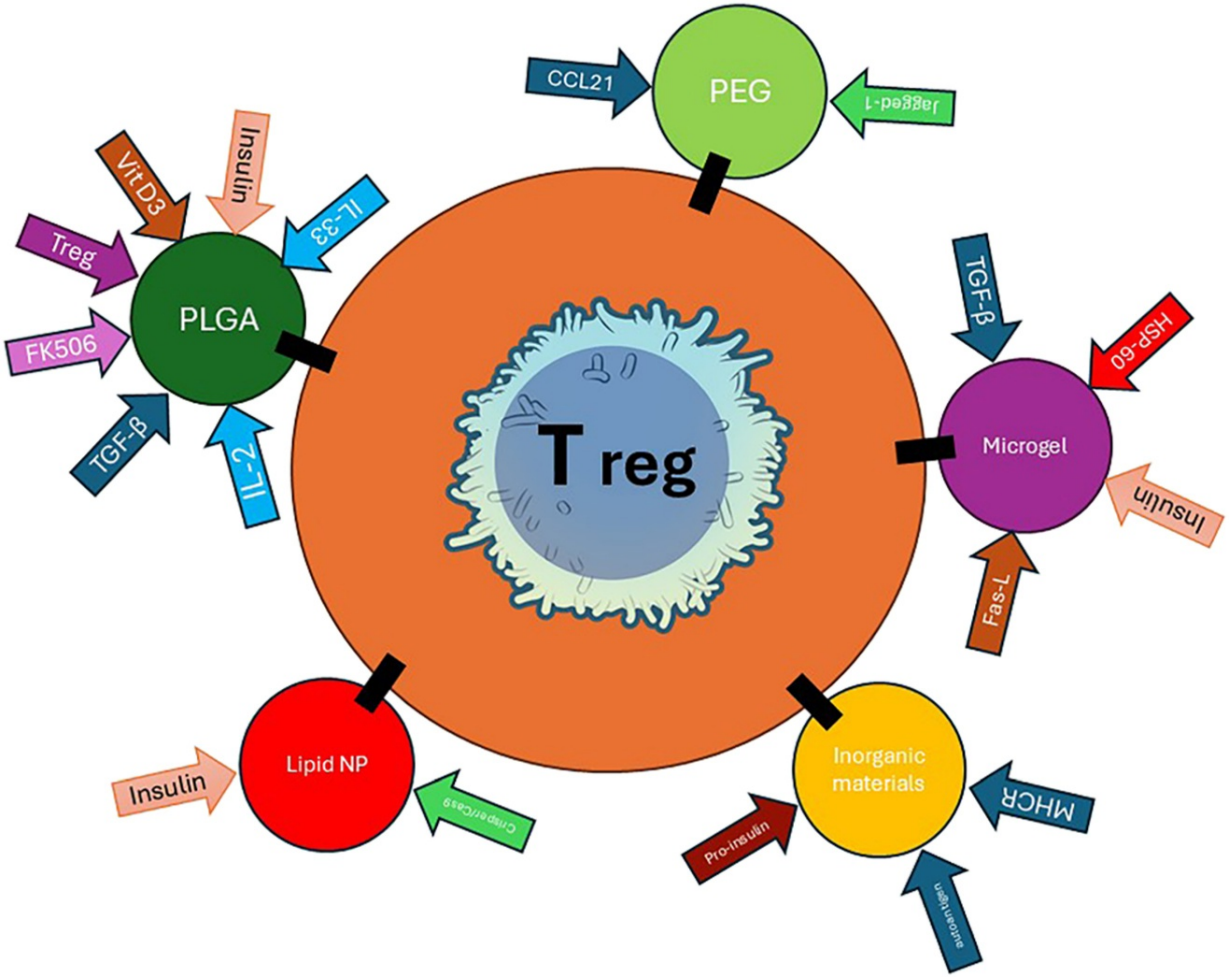
Tolerance and biomaterials. This schematic Figure shows the integration of biomaterials, such as PLGA, with immunomodulatory agents used to target T*reg*, which plays a pivotal role in tolerance.

From a personalised medicine perspective, antigen‐specific tolerance induction represents a promising frontier, enabling indefinite graft acceptance with minimal systemic immunosuppression [220, 263]. T*regs* play a central role in sustaining self‐tolerance through cell–cell interactions and immunosuppressive cytokines (e.g., IL‐10, TGF‐β1). However, the studies performed to induce tolerance in the condition of transplantation of islets of Langerhans are shown in Table 4 and Figure 4. Conventional immunosuppressive agents are often inadequate in suppressing memory T cell responses, leaving transplanted grafts vulnerable. To address this limitation, biomaterials are being engineered to mitigate antigen shedding and the release of danger‐associated signals, thereby reducing alloreactivity in a patient‐tailored manner. In parallel, strategies that integrate biomaterial scaffold T*reg*‐centred immunotherapies are emerging, providing localised support for T*reg* function and reinforcing long‐term graft tolerance [287]. These hybrid strategies align strongly with the preventive and personalised axes of 3 PM.

**TABLE 4:** Advanced biomaterial‐based and immunomodulatory strategies for enhancing islet transplantation tolerance and long‐term graft survival in type 1 diabetes.

#	Studies	Study model	Results
1	Poly(lactic‐co‐glycolic) acid (PLGA) + FK506	Mice	The islets co‐delivered with 10 mg/kg FK506 maintained normal blood glucose levels (survival rate: 60%). Also, a minimal number of immune cells invaded the gel of the islet‐FK506 group [264].
2	Poly(lactide‐co‐glycolide) + Treg	Mice	PLG scaffolds can induce long‐term graft survival in an autoimmune diabetes model [220].
3	Microgel + FasL	Mice	Localised immunomodulation results in prolonged survival of allogeneic islet grafts in diabetic mice [263].
4	Microgel + streptavidin (SA)/programed cell death‐1 + biotin + FasL	Mice	This scaffold improves local retention of the immunomodulatory agent over 3 weeks in vivo and increases T*reg* and anergic cells [265].
5	Liposome + insulin peptide	Mice	Phosphatidylserine‐liposomes loaded with insulin peptides induce tolerogenic dendritic cells and impair autoreactive T cell proliferation that results in arresting the autoimmune aggression, reducing the severity of insulitis, and preventing type 1 diabetes (*T1D*) by apoptotic mimicry [256].
6	PLGA + TGF‐B	Mice	Transplantation of islets into diabetic mice on TGF‐B1 scaffolds improved the ability of syngeneic islets to control blood glucose levels within the first week of transplant and delayed rejection of allogeneic islets [116].
7	Alginate + TGF‐B	Balb/c mice to C57BL/6 mice	These factors create the necessary foundation for the device to develop vascularisation and induce strong and localised tolerance specific to allogeneic cells, which subsequently triggers tolerance in peripheral T‐cells [266].
8	PLGA + IL‐10	Mice	*IL‐10* gene delivery decreased inflammation by modulating cytokine expression of infiltrating leucocytes [267].
9	PLGA + IL‐33	Mice	Delivering IL‐33 locally enhances the presence of T*regs* in adipose tissue and diminishes populations of T cells that can damage the graft. However, it may also stimulate innate cell populations, which could hinder the process of cell engraftment [108].
10	Polyethylene glycol (PEG) + Jagged (JAG)‐1	Rat to mice	JAG‐1 immobilisation could improve immunoprotection of pancreatic islets by localised modulation of the immune milieu from an inflammatory to an anti‐inflammatory state [141].
11	Alginate + CXCL‐12	Mice	This chemokine resulted in long‐term allo‐ and xeno‐islet survival and function, as well as a selective increase in intra‐graft T*regs* [117].
12	PEG + PLGA + Lipid + CRISPR/Cas9	Balb/c mice to C57BL/6 mice	CLAN (mCas9/gCD40)‐mediated CD40 disruption inhibited T cell activation, which reduced graft damage and prolonged graft survival [268].
13	Iron oxide + MHC peptide	Nude mice	Nanoparticles coated with human diabetes‐relevant pHLA complexes restored normoglycemia in a humanised model of diabetes [269]. In another study, these nanomedicines promote the differentiation of disease‐primed autoreactive T cells into TR1‐like cells, which in turn suppress autoantigen‐loaded antigen‐presenting cells and drive the differentiation of cognate B cells into disease‐suppressing regulatory B cells, without compromising systemic immunity [270].
14	PLGA + TGF‐B + granulocyte‐macrophage colony‐stimulating factor (GM‐CSF) + Vit D3	Mice	This scaffold protected 40% of mice from *T1D* development [271].
15	PLGA + vitD3 + insulin/TGF‐B + GM‐CSF	Mice	This PLGA MP system prevented diabetes onset in 60% of nonobese diabetic (NOD) mice [272].
16	PLGA + insulin + CpG hydrogel + GM‐CSF	Mice	This hydrogel (GM‐CSF/CpG)/insulin‐MP vaccine protected 40% of NOD mice from *T1D*. Also, CpG stimulation increased IL‐10 production as an anti‐inflammatory cytokine [273].
17	PLGA + alginate + GM‐CSF + BDC peptide	NOD mice	This non‐inflammatory biomaterial system can generate antigen‐specific T*regs* in vivo and does not accelerate diabetes [274].
18	Acetylated dextran (Ace‐DEX) + rapamycin + diabetogenic peptide P31	NOD mice	Ace‐DEX microparticles‐based antigen‐specific therapy efficiently induces tolerance in diabetogenic CD4^+^ T cells, thereby preventing *T1D* [275].
19	PEG + CCL21	NOD mice	Sustained release of β‐cell antigens and CCL21 immunomodulatory molecules could enable the development of antigen‐specific tolerance therapies for *T1D* [276].
20	NAP hydrogel + GFFY peptide	NOD mice	Inoculation with Nap‐GFFY fully protects NOD mice from developing *T1D* up to 36 weeks. It preserves pancreatic islet structure with minimal immune infiltration, primarily by boosting peripheral T*regs* and maintaining circulating TGF‐β1 levels [277].
21	PLGA + anti CD4+ IL‐2 + TGF‐B + rapamycin	Mice T cell isolation	Controlled release formulations of IL‐2, TGF‐β, and rapamycin can induce functional T*reg* in vitro with the potential to be developed into an in vivo T*reg* induction and expansion therapy [278].
22	Gold nanoparticle (NP) + ITE + proinsulin	Mice	Aryl hydrocarbon receptor (AHR) regulates both T*reg* and TH17 cell differentiation in a ligand‐specific fashion, constituting a unique target for therapeutic immunomodulation [279]. The endogenous AHR ligand ITE helps induce active immunologic tolerance by directly affecting DCs and T cells, highlighting nontoxic endogenous AHR ligands as promising compounds for treating autoimmune disorders [280]. NP_ITE+Ins_ triggered a tolerogenic phenotype in DCs, marked by a reduced capacity to activate inflammatory T cells and an increased differentiation of FoxP3+ T*reg* cells. This shift, driven by AHR‐dependent Socs2 induction, inhibited NF‐κB activation and proinflammatory cytokine production. These findings indicate that NPs could help restore tolerance in *T1D* and other autoimmune disorders [281].
23	PLGA + polystyrene beads + CD40/80/86	NOD mice	Dendritic cells from the spleens of microsphere‐treated mice show reduced expression of cell surface markers CD40, CD80, and CD86. This innovative microsphere formulation is the first nucleic acid vaccine to suppress and reverse diabetes by inducing an immunoregulatory phenotype in endogenous dendritic cells [282].
24	SA + FasL + rapamycin	Mice	SA‐FasL‐engineered islet grafts successfully restored normal blood glucose levels in chemically induced diabetic syngeneic mice. Additionally, transplanting SA‐FasL‐engineered BALB/c islet grafts along with a brief rapamycin treatment induced strong localised tolerance in all C57BL/6 recipients [283, 284].
25	PLGA + LIF + PEG + islet	Mice	The average survival time of islets coated with FK506/DOPA‐NPs was considerably longer compared to those coated with DOPA‐NPs without FK506 and the control group [285].
26	PLGA + COUR NPs (CNP)	NOD mice	Treatment of NOD mice with CNPs containing a single protein suppressed the corresponding Ag‐specific T‐cell response. However, the prevention of overt *T1D* was only achieved when all three diabetogenic proteins were included within the CNPs (CNP‐*T1D*). This blockade of *T1D* through CNP‐T1D tolerisation led to the induction of T*reg* cells and a notable reduction in both peri‐insulitis and immune cell infiltration into the pancreatic islets [286].

In summary, the convergence of biomaterials and immunomodulatory agents within the 3 PM paradigm provides a powerful path towards durable immune tolerance in islet transplantation. While significant progress has been achieved, future directions should emphasise predictive biomarker‐driven patient stratification, preventive immune regulation at early graft stages, and the personalisation of tolerance‐inducing strategies. Such an integrated approach holds promise to transform islet transplantation from experimental therapy into a clinically reliable and individualised solution for *T1D*.

## 2 Conclusion

Advancements in transplantation technologies are revolutionising the management of *T1D*, extending beyond conventional insulin therapy towards true β‐cell replacement and restoration of physiological glucose regulation. Islet allotransplantation, xenotransplantation, and encapsulation systems continue to demonstrate significant therapeutic promise; however, immune rejection, fibrotic encapsulation, and limited graft longevity remain major clinical challenges. Optimising biomaterials to balance immune protection with adequate nutrient and oxygen permeability is therefore fundamental to advance transplantation efficacy. Within the conceptual and practical framework of 3 PM, future progress in transplantation should embrace a systems‐level and patient‐centred approach. First, predictive modelling, informed by immune phenotyping, metabolic profiling, and bioenergetic biomarkers, should be developed to identify optimal candidates for transplantation and forecast graft survival dynamics. Second, preventive immunomodulation strategies, including the local delivery of tolerogenic factors, antioxidant compounds, and mitochondrial support agents, can mitigate early graft inflammation and systemic toxicity. Third, personalised graft design, supported by 3D bioprinting and adaptive biomaterials, enables individualised tuning of pore architecture, bioink composition, and immunomodulatory payloads to align with each patient's unique immune and metabolic profile.

The integration of AI and digital health monitoring provides an essential dimension to this transformation. AI‐driven analytics can synthesise multi‐modal data, from omics and imaging to wearable sensors, to generate predictive digital twins that simulate graft performance and optimise clinical decision‐making in real time. Such data‐centric approaches will help bridge the gap between laboratory research and patient‐level personalisation, enabling continuous monitoring and adaptive interventions.

Ultimately, achieving durable graft tolerance with minimal systemic immunosuppression demands a holistic, 3 PM‐aligned paradigm that unites biomaterial science, immunoengineering, and regenerative medicine. By embedding predictive diagnostics, preventive interventions, and personalised design principles into transplantation research, the field can transition from reactive glucose management tos anticipatory, individualised regenerative therapy. This integrative strategy holds the potential to ensure long‐term metabolic stability, enhanced quality of life, and sustainable therapeutic outcomes for patients with *T1D*.

## Author Contributions

F. Sabet Sarvestani, M. Golshan, and M. H. Karimi conceived and designed the study. R. Yaghobi, M. Khosravi Maharlooei, and M. H. Karimi developed the theoretical framework. F. Sabet Sarvestani draughted the manuscript. F. Sabet Sarvestani, R. Yaghobi, M. Khosravi Maharlooei, and M. H. Karimi critically reviewed and revised the manuscript for important intellectual content. All authors have read, approved, and agreed to the published version of the manuscript.

## Funding

The authors have nothing to report.

## Conflicts of Interest

The authors declare no conflicts of interest.

## Data Availability

The data that support the findings of this study are available from the corresponding author upon reasonable request.
